# Eosinophilic dermatosis of hematologic malignancy associated with angioimmunoblastic T-cell lymphoma

**DOI:** 10.1016/j.jdcr.2025.08.038

**Published:** 2025-09-19

**Authors:** Jamie B. Harris, Arthur M. Samia, Christina W. Sun, Kiran Motaparthi

**Affiliations:** a University of Florida College of Medicine, Gainesville, Florida; b Department of Dermatology, University of Florida, Gainesville, Florida

**Keywords:** eosinophils, exanthema, hematologic neoplasms, lymphoma, peripheral, pruritus, T-cell

## Introduction

Eosinophilic dermatosis of hematologic malignancy (EDHM) is a reactive cutaneous phenomenon most frequently associated with chronic lymphocytic leukemia (CLL) and often presents as a persistent arthropod bite-like reaction.^1^ Angioimmunoblastic T-cell lymphoma (AITL), a rare form of non-Hodgkin lymphoma (NHL), is associated with poor prognosis and may be heralded by a wide variety of cutaneous manifestations.^2^ Management ranges from systemic corticosteroids to targeted therapy, chemotherapy, and autologous stem cell transplant.^3^ We describe EDHM as the presenting sign of AITL.

## Case report

**Fig 1:**
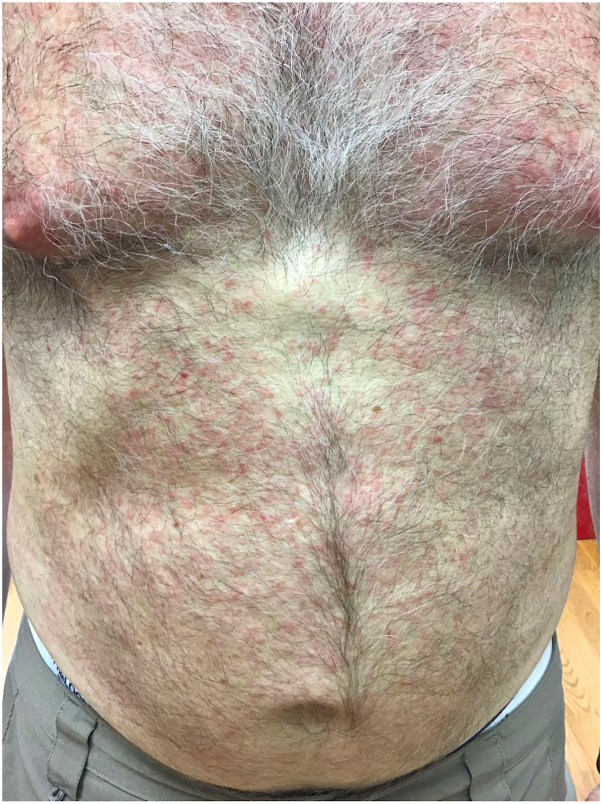
Diffuse papular eruption on the chest and abdomen.

**Fig 2:**
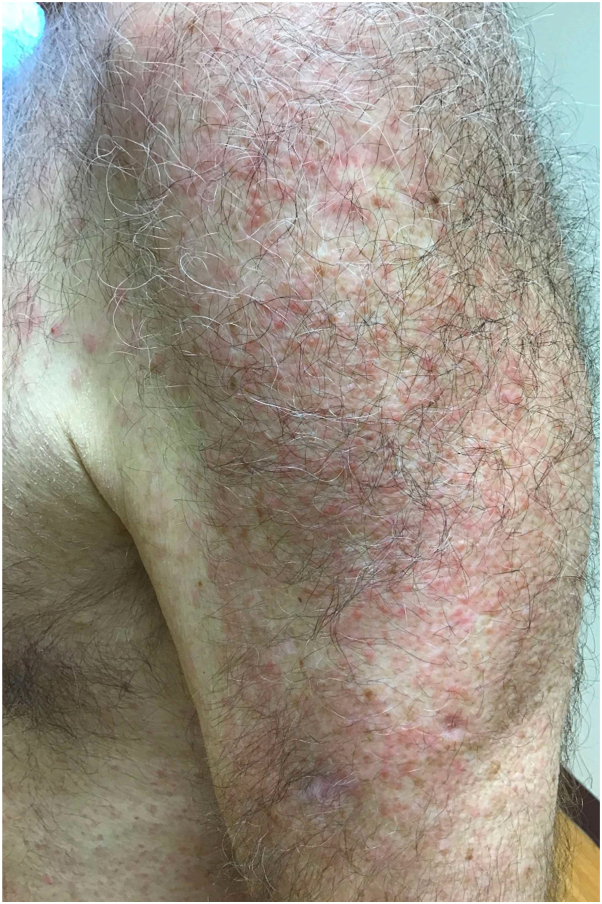
Diffuse erythematous papules on the shoulder and arm.

A 69-year-old man with no significant oncologic history presented with a 2-month history of a markedly pruritic rash involving the face and trunk. He also reported intermittent fevers and arthralgias during this time. Physical examination revealed an erythematous papular eruption involving the face, trunk, and upper extremities but with minimal involvement of the lower extremities (Figs 1 and 2).

**Fig 3:**
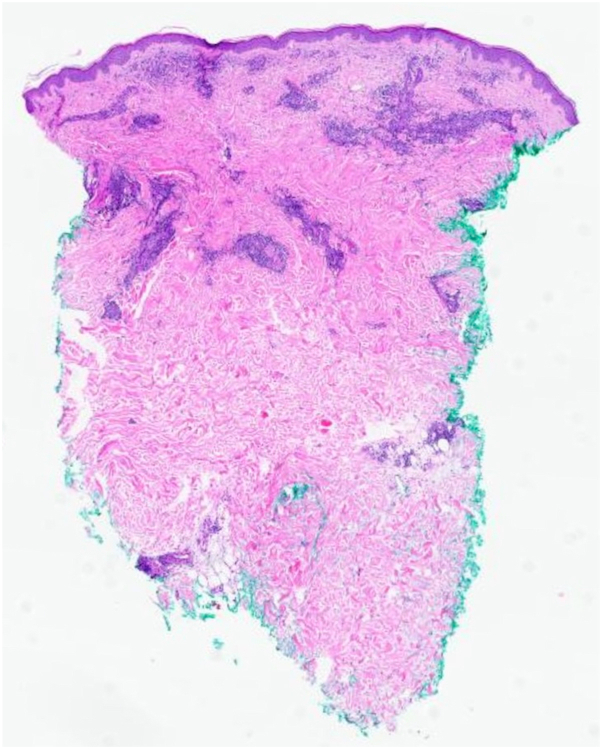
Arthropod bite-like infiltrate, with mild dermal edema and a superficial and deep perivascular lymphocytic infiltrate with numerous eosinophils and plasma cells (hematoxylin & eosin, 20× magnification).

**Fig 4:**
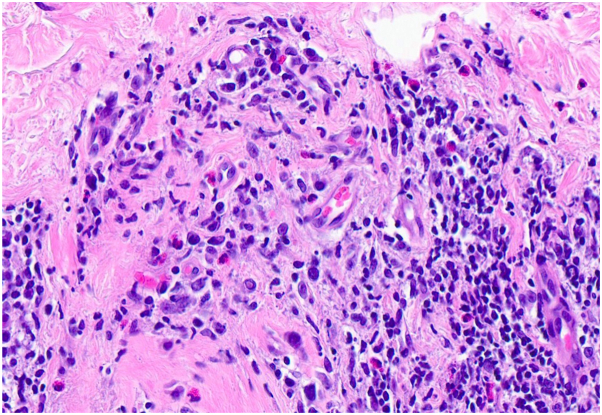
Lymphocytic infiltrate with numerous eosinophils (hematoxylin & eosin, 200× magnification).

He was previously treated for a presumed drug-induced hypersensitivity reaction due to omeprazole with a prednisone taper and elimination of nonessential medications; however, the rash did not resolve, and at follow-up several weeks later, the patient reported worsening pruritus. Punch biopsies demonstrated a superficial and deep perivascular infiltrate with abundant eosinophils (Figs 3 and 4).

Dermal hypersensitivity reaction, drug eruption, EDHM, and papular urticaria were among the primary considerations in the initial clinical differential diagnosis of this intensely pruritic eruption. Although the arthropod bite-like histological pattern was suggestive of papular urticaria, the clinical findings were not consistent with bite reaction. The lack of improvement following discontinuation of nonessential medications made drug eruption less likely. The patient’s advanced age, prominent constitutional symptoms, and distribution predominantly above the waist pointed toward EDHM as the most likely diagnosis. Polymerase chain reaction studies for T-cell receptor gene rearrangement were attempted on the skin biopsy but could not be performed due to insufficient tissue.

A complete blood count with differential demonstrated an increased absolute eosinophil count of 1.1 × 10^9^/L (reference range: 0.02-0.5 × 10^9^/L). The patient was referred to a hematologist for additional evaluation and exclusion of underlying leukemia or lymphoma. Flow cytometry disclosed peripheral blood eosinophilia and a minor clonal population of dim CD20-positive B lymphocytes co-expressing CD5 and CD23, indicative of CLL-type monoclonal B-cell lymphocytosis (MBL). Bone marrow biopsy with flow cytometry excluded CLL. Computed tomography of the chest, abdomen, and pelvis demonstrated extensive retroperitoneal and axillary lymphadenopathy and mild superior mediastinal lymphadenopathy.

To allow tapering of prednisone and to address the marked pruritus, dupilumab 300 mg subcutaneously every other week was initiated. Two months after the initial presentation, the patient’s rash and pruritus were significantly improved. However, shortly after, he developed diffuse lymphadenopathy. An excisional axillary lymph node biopsy demonstrated pathology characteristic for AITL: a polymorphic inflammatory cell infiltrate with increased paracortical blood vessels. Flow cytometry revealed aberrant T-cell CD10 expression and CD3 positivity on the majority of cells. Fluorescence *in situ* hybridization revealed Epstein-Barr encoding region-positive immunoblasts. Polymerase chain reaction testing was positive for clonal rearrangements of T-cell receptor (TCR)-beta and TCR-gamma genes. He was referred to a specialty cutaneous lymphoma clinic for further evaluation, and prednisone was reinitiated with the goal of inducing partial remission of AITL. Four months after initial presentation, ongoing treatment with dupilumab and prednisone 10 mg daily resulted in clearance of his rash. One month later, after the patient was evaluated by an oncology service, this regimen was discontinued. The patient was started on definitive treatment for AITL with brentuximab vedotin, cyclophosphamide, doxorubicin, and rituximab with a planned autologous hematopoietic stem cell transplant.

## Discussion

EDHM was first described as recalcitrant arthropod bite-like reactions in patients with known hematological malignancies.^4^ Currently, the term “EDHM” encompasses a variety of clinical presentations, including eosinophilic folliculitis, that share histopathologic findings. EDHM may precede the diagnosis of an underlying hematologic malignancy by months to years.^5^

EDHM presents as a polymorphic, pruritic eruption of papules, nodules, urticarial plaques, vesicles, or bullae.^4^ Lesions most commonly occur above the waist with relative sparing of the lower extremities. The diagnosis can be made based on these clinical features in conjunction with histopathology showing eosinophil-rich superficial and deep dermal lymphocytic infiltrate.^1^ Other key findings may include eosinophilic spongiosis and the presence of eosinophils within the folliculosebaceous unit. Over 200 cases have been reported in the literature with the majority associated with CLL (76.7%), followed by mantle cell lymphoma (4.3%) and acute leukemia (1.9%).^4^ Although we initially considered CLL as a possible underlying malignancy, bone marrow biopsy did not support this diagnosis. MBL can progress to CLL at an annual reported rate between 1% and 5%; however, an association between MBL and EDHM has not previously been described. Given that MBL can be found in approximately 20% of patients over the age of 70 years, the presence of MBL in this patient was likely an unrelated incidental finding. Two prior cases of EDHM have been associated with T-cell lymphoma, including cutaneous T-cell lymphoma and aggressive T-cell lymphoma.^1^^,^^6^ Although cutaneous involvement of AITL cannot be entirely excluded given that immunohistochemistry was not performed on the initial biopsy, the patient’s clinical course and histopathologic findings support the diagnosis of EDHM associated with AITL.

AITL makes up 1.2% of NHL and 18% of peripheral T-cell lymphomas and follows an aggressive clinical course.^7^ AITL is diagnostically challenging, complicated by its variable clinical presentations, including generalized lymphadenopathy, B-symptoms, effusions, and, in half of cases, cutaneous manifestations.^3^ AITL is associated with specific and nonspecific skin findings. Nonspecific findings include purpura, petechiae, and erythroderma. Specific findings include chronic morbilliform eruptions, which often mimic viral exanthems or drug eruptions.^2^ The diagnosis can be definitively made through characteristic histopathology, immunophenotyping on lymph node biopsy, and TCR gene rearrangement studies.^3^ Histopathologic features observed in specific infiltrates (cutaneous involvement by AITL) include high endothelial venules, reactive B cell infiltrates, Epstein-Barr virus (EBV)-positive B cells, and atypical T cells with a T follicular helper cell immunophenotype, including expression of PD-1, CXCL13, CD10, and BCL6.^2^^,^^8^ Additionally, lymph node biopsy may show EBV-infected cells and atypical T cells with a T follicular helper phenotype.^2^^,^^8^ The absence of these features makes a diagnosis of AITL unlikely and instead supports a reactive (nonspecific) infiltrate. Early recognition of EDHM in this case led to prompt workup and diagnosis of AITL. Given that clinical features of morbilliform eruptions may reflect either neoplastic (specific) or reactive (nonspecific) infiltrates in this setting, it is possible that some previously reported cases of AITL with morbilliform clinical appearance may have represented EDHM secondary to AITL, particularly in the absence of skin biopsy with diagnostic features of AITL.

EDHM is primarily managed by treating the underlying malignancy.^4^ For refractory cases, dupilumab has demonstrated efficacy.^9^ EDHM may be driven by an immune shift toward a T helper 2-associated response.^9^

However, dupilumab has also been associated with the “unmasking” of undiagnosed cutaneous T-cell lymphomas, driven by increased IL-13Rα2 signaling activating tumor proliferation and immune evasion, resulting in generalized lymphadenopathy and B-symptoms after initiation of dupilumab.^10^ AITL has also been unmasked following dupilumab treatment for atopic dermatitis.^11^ This mechanism appears less likely in the presented case. This patient experienced significant clinical improvement of skin lesions while on dupilumab, arguing against cutaneous involvement of AITL. In the patient presented here, we suspect that tapering systemic corticosteroids, and thereby removing immunosuppressive control, unmasked underlying AITL.

Given that it may be the presenting sign of a lymphoproliferative disorder, EDHM should be considered in elderly patients who present with papular pruritic eruptions characterized by numerous eosinophils on histopathology. As a nonspecific manifestation, EDHM can herald the onset of AITL, permitting early recognition and prompt treatment. While dupilumab is effective for EDHM, close monitoring is warranted given the potential for unmasking an underlying lymphoma.

## Conflicts of interest

None disclosed.
